# Risk factors for impaired renal function in HIV-infected and HIV-uninfected adults: cross-sectional study in North-Western Tanzania

**DOI:** 10.1186/s12882-021-02563-z

**Published:** 2021-10-29

**Authors:** Bazil Baltazar Kavishe, Belinda V. Kweka, Dorothea Nitsch, George PrayGod, Kidola Jeremiah, Daniel Faurholt-Jepsen, Suzanne Filteau, Mette Frahm Olsen, Brenda W. Kitilya, Rikke Krogh-Madsen, Henrik Friis, Robert Peck

**Affiliations:** 1 Mwanza Research Centre, National Institute for Medical Research, Mwanza, Tanzania; 2 Faculty of Epidemiology and Population Health, London School of Hygiene & Tropical Medicine, London, UK; 3 Department of Infectious Diseases, Rigshospitalet, Copenhagen, Denmark; 4 Department of Nutrition, Exercise and Sports, University of Copenhagen, Copenhagen, Denmark; 5 Centre for Physical Activity Research, Copenhagen University Hospital – Rigshospitalet, Copenhagen, Denmark; 6 Department of Infectious Diseases, Copenhagen University Hospital, Hvidovre, Copenhagen, Denmark; 7 Mwanza Intervention Trials Unit/National Institute for Medical Research, Mwanza, Tanzania; 8 Weill Cornell Medical College, New York, USA

**Keywords:** Estimated glomerular filtration rate, Impaired renal function, HIV, Antiretroviral therapy

## Abstract

**Background:**

Although the burden of impaired renal function is rising in sub-Saharan Africa (SSA), little is known about correlates of impaired renal function in the region. We determined factors associated with estimated glomerular filtration rate (eGFR) and impaired renal function in HIV-infected and HIV-uninfected adults.

**Methods:**

We undertook cross-sectional analysis of data from 1947 adults at enrolment for a cohort study on diabetes and associated complications in HIV patients in Mwanza, north-western Tanzania. A structured questionnaire was used to collect data on sociodemography, smoking, alcohol, physical activity, antiretroviral therapy (ART) and anthropometry. We measured blood pressure, tested blood samples for creatinine, glucose and HIV, and performed Kato Katz for *Schistosoma mansoni*. Correlates of eGFR (mL/min/1.73 m^2^) and impaired renal function (eGFR< 60 mL/min/1.73 m^2^) were determined using linear regression and logistic regression, respectively.

**Results:**

655 (34%) participants were HIV-uninfected, 956 (49%) were ART-naive HIV-infected and 336 (17%) were HIV-infected adults on ART. The mean age was 41 years (SD12) and majority (59%) were females. Overall, the mean eGFR was 113.6 mL/min/1.73 m^2^ but 111.2 mL/min/1.73 m^2^ in HIV-uninfected, 109.7 mL/min/1.73 m^2^ in ART-naive HIV-infected and 129.5 mL/min/1.73 m^2^ in HIV-infected ART-experienced adults, and respective prevalence of impaired renal function was 7.0, 5.7, 8.1 and 6.3%. Correlates of lower eGFR were increasing age, higher socioeconomic status, unhealthy alcohol drinking, higher body mass index and diabetes mellitus. Anaemia was associated with 1.9 (95% Confidence Interval (CI):1.2, 2.7, *p* = 0.001) higher odds of impaired renal function compared to no anaemia and this effect was modified by HIV status (*p* value 0.02 for interaction).

**Conclusion:**

Impaired renal function is prevalent in this middle-aged study population. Interventions for prevention of impaired renal function are needed in the study population with special focus in HIV-infected adults and those with high socioeconomic status. Interventions targeting modifiable risk factors such as alcohol and weight reduction are warranted.

**Supplementary Information:**

The online version contains supplementary material available at 10.1186/s12882-021-02563-z.

## Background

The growing epidemic of non-communicable disease (NCD) in sub-Saharan Africa (SSA) poses a significant burden to the health care system in the region where prevalence of infectious diseases particularly human immunodeficiency virus (HIV) is still very high [1]. The relative contribution of impaired renal function to the global burden of disease significantly has increased with Disability-Adjusted Life Years attributable to chronic kidney disease rising by 42% between 1990 and 2017 [2, 3]. Economic consequences related to impaired renal function are enormous [4] calling for urgent intervention measures.

Both infectious and non-infectious risk factors have been associated with impaired renal function in SSA. In this region where two thirds of the HIV-infected population lives [5], impaired renal function is projected to rise as consequences of the direct insult of HIV virus on the kidneys or adverse effects of antiretroviral therapy (ART) [6]. In addition, schistosomiasis is highly prevalent in SSA and has been associated with impaired renal function in many epidemiological studies [7]. Moreover, the rising lifestyle risk factors for NCD in SSA [1] could fuel impaired renal function to epidemic proportions in the near future [3]. Unlike high income countries where impaired renal function is more common in middle-aged and elderly people, and mainly driven by diabetes mellitus and associated with hypertension, impaired renal function in SSA occurs in relatively younger people and is mainly associated with hypertension and/or glomerulonephritis [8].

If recent genetic evidence is correct [9], hypertension may well be the first sign of underlying renal disease, and the context of onset of hypertension in a relatively young population in SSA then warrants a closer examination of kidney function markers. A high prevalence of hypertension and lifestyle risk factors for NCDs has been reported in north-western Tanzania and southern Uganda and a substantial proportion of young adults had hypertension [10, 11]. Other risk factors for impaired renal function common in SSA such as HIV infection and schistosomiasis may play an important role but research data are limited. Few high quality studies have been conducted to determine the burden and risk factors for impaired renal function in SSA and majority of these studies used measurement of urine protein to assess renal function [12, 13]. In a population-based survey in north-western Tanzania, increasing age, physical inactivity and higher blood pressure were associated with lower estimated glomerular filtration rate (eGFR) but this study was not designed with sufficient power to determine the effect of HIV on impaired renal function and the role of schistosomiasis was not investigated [14]. High quality epidemiological studies using eGFR to estimate renal function are therefore needed to inform preventive measures tailored to curb prevailing risk factors for impaired renal function in SSA.

The aim of this study was to determine factors associated with eGFR and impaired renal function in HIV-infected and HIV-uninfected adults. We hypothesised that diabetes mellitus, hypertension and schistosomiasis are associated with low eGFR and that HIV infection modifies these associations.

## Methods

### Study design and population

We conducted a cross-sectional study using data collected at enrolment visit for a cohort study on diabetes and associated complications in HIV patients in Mwanza, north-western Tanzania locally referred to as the Chronic Infections, Co-morbidities and Diabetes in Africa (CICADA) study, registered at https://clinicaltrials.gov as NCT03106480.

HIV-infected and HIV-uninfected adults (≥ 18 years) from three pre-determined groups were enrolled from October 2016 to November 2017. The first group includes adults from two previous studies in Mwanza: Nutrition, Diabetes and Pulmonary Tuberculosis (TB-NUT), and Nutritional Support for African Adults Starting Antiretroviral Therapy (NUSTART). TB-NUT registered at https://clinicaltrials.gov as NCT00311298 was conducted from 2006 to 2009 [15–18] while NUSTART registered as PACTR201106000300631 at Pan African Clinical Trial registry was undertaken from 2011 to 2013 [19]. All HIV-infected participants from these previous studies were already on ART. The second group of participants were HIV-infected ART-naïve adults from HIV care and treatment clinics in Mwanza, and the third group were HIV-uninfected adults from the residential neighbourhood of enrolled ART-naïve HIV-infected participants.

### Recruitment of study participants

Participants’ recruitment procedures have been described in detail elsewhere [20]. Based on survival status at the end of TB-NUT and NUSTART, individuals who were known to be alive were invited to join CICADA study through telephone contact or home visit. HIV-infected adults who were about to start ART were identified from HIV care and treatment clinics in Mwanza and invited to join the study. A computer-generated random list of 50% enrolled ART-naïve HIV-infected adults was used for random selection of HIV-uninfected adults for enrolment into the study. As described previously [20], a field worker visited the street/sub-village where the index HIV-infected adult lives. With the help of the street/sub-village leader, a complete list of households in the area was prepared. Thereafter, we randomly selected three households which were visited one by one until an eligible adult was found. In a situation where we did not find eligible adult in the first list of randomly selected households, the process was repeated until an eligible participant was enrolled.

Potential participants were eligible to join the study if they were ≥ 18 years, resident in the study area and not planning to relocate within 3 years after enrolment. In addition, for ART naïve HIV-infected adults they should have been willing to start ART immediately after study enrolment. HIV-uninfected adults were enrolled if lived in the same street/sub-village, of same sex, within 5 years of age of the index HIV-infected adult, and tested negative for HIV using rapid tests as part of the study procedures. Participants were excluded if they were pregnant or terminally ill.

After assessment for eligibility, potential participants were instructed to fast overnight on a scheduled study clinic appointment date for informed consent and possible study enrolment. One day before scheduled enrolment visit, potential participants were contacted by telephone and reminded to come the following day after an overnight fast. Consenting participants were enrolled and underwent study procedures as described below.

### Data collection

As previously described [20] we used a structured questionnaire adapted from STEPwise approach to chronic disease risk factor surveillance (STEPS) tool [21] to collect data on socio demographic variables (i.e. age, sex, education), and risk factors for NCDs including physical inactivity, alcohol use and smoking. A clinical questionnaire was used to capture information on previous history of diabetes and hypertension and drug use for these conditions, and information on ART use was retrieved from participants’ clinical records.

We took three measurements for weight using digital scale and height using a stadiometer (Seca, Germany) and used their median values to calculate body mass index (BMI) as weight (kg)/(height(m))^2^. Using a body composition bio-impedance analyser (Tanita BC418, Tokyo, Japan), we measured fat mass (kg) and fat-free mass (kg) and computed fat mass index as fat mass (kg)/(height(m))^2^ and fat-free mass index as fat-free mass (kg)/height (m))^2^. With the participant seated and the arm supported at the level of the heart, we used a digital blood pressure monitor (Omron Health Care Manufacturing Vietnam Co., Ltd., Binh Duong Province, Vietnam) with small, medium, or large cuff depending on mid upper arm circumference to measure blood pressure three times. We first measured blood pressure once on each arm and took a third measurement on the arm with highest blood pressure reading. The average of the three blood pressure readings was used in the analysis.

On arrival at the study clinic, and after confirmation of fasting status, we collected blood sample for serum creatinine, full blood count, blood glucose and HIV testing (for participants with unknown HIV status), and stool samples for *Schistosoma mansoni* eggs. Participant underwent a standard oral glucose tolerance test (OGTT). After collection of fasting blood glucose sample, participants were asked to drink (within 5 min) a solution containing 75 g of glucose in 250 mL of drinking water. Another blood sample for glucose measurement was collected 120 min after oral administration of glucose solution. Blood glucose was measured at the study clinic using Hemocue Glucose 201 RT machine (Hemocue AB, Angelholm, Sweden). Laboratory samples for other tests were transported in a cool box to the National Institute for Medical Research (NIMR) laboratory in Mwanza within 2 h of collection for testing. NIMR laboratory participates in external quality assurance programs including the Royal College of Pathologists of Australasia Quality Assurance Program.

Serum creatinine was performed using A25 biochemistry analyser (Biosystems S.A; Costa Brava, 30; 08030 Barcelona; Spain) with modified Jaffe method traceable to isotope dilution mass spectrometry and full blood count was measured using Beckman Coulter AcT5diff AL haematology analyser (Beckman Coulter Inc. 11,800 SW 147 Avenue, Miami, Florida 33,196–2500 USA). HIV tests were performed according to national guidelines using SD Bioline (SD HIV- 1/2 3.0SD standard diagnostics Inc) and Unigold (The Uni-Gold, Trinity Biotech, IDA Business Park, Bray, Co. Wicklow, Ireland) rapid tests. Discordant results were analysed using the Uniform II vironostika-HIV Ag/Ab Micro-Elisa system (Biomerieuxbv, The Netherlands). *Schistosoma mansoni* eggs were identified using Kato Katz technique.

### Definitions

We measured renal function by calculating eGFR using the Chronic Kidney Disease Epidemiology (CKD-EPI) collaboration formula without a race correction factor since this correction factor has been reported to overestimate eGFR in African populations [22, 23]. Impaired renal function was defined according to Kidney Disease Improving Global Outcome (KDIGO) guidelines [24] as eGFR < 60 mL/min/1.73m^2^. We further categorised renal function according to KDIGO guidelines as follows: normal (≥ 90), mildly decreased (60–89), mildly to moderately decreased (45–59), moderately to severely decreased (30–44), severely decreased (15–29) and kidney failure (< 15). Renal hyperfiltration was defined as eGFR > 135 mL/min/1.73m^2^ [25]. BMI (kg/m^2^) was categorised into three groups as underweight (< 18.5), normal weight (18.5 - < 25) and overweight/obesity (≥ 25). Anaemia was defined according to the World Health Organization (WHO) criteria as haemoglobin level < 12 mg/dL for women and < 13 mg/dL for men [26]. Hypertension was defined as systolic blood pressure ≥ 140 mmHg and/or diastolic blood pressure ≥ 90 mmHg or on medication for hypertension. Diabetes mellitus was defined according to WHO guidelines [27] as 2 h oral glucose tolerance test blood glucose level ≥ 11.1 mmol/L or on medication for diabetes. Unhealthy alcohol drinking was defined as habitual alcohol drinking of more than two standard drinks/day for women or more than three standard drinks/day for men.

### Data analysis

Data was analysed using Stata 14 data analysis software (College Station, Texas, USA). Participants’ background characteristics were described using frequencies and proportions stratified by HIV status. Prevalence of impaired renal function and eGFR grades were also described using frequencies and proportion.

We used multivariable linear regression to assess the association between covariates with both eGFR as a continuous outcome (primary analysis) and impaired renal function defined by eGFR < 60 mL/min/1.73m^2^ (secondary analysis). All analyses were adjusted for age, sex and fat-free mass a priori. As participants may have very low muscle mass, which may lead to an overestimation of renal function by eGFR, we adjusted fat-free mass a priori. We used interaction terms for effect modification in multivariable linear regression to test for effect modification by HIV status for all exposure variables. For any exposure variables with evidence of statistically significant effect modification by HIV status, we then determined the association between exposure variables and eGFR stratified by HIV status. In addition, we used logistic regression to determine factors associated with impaired renal function (eGFR < 60 mL/min/1.73m^2^) following similar steps as for linear regression. Associations were considered statistically significant if the *p* value was < 0.05.

### Ethical considerations

The study was approved by the Medical Research Coordinating Committee of the Tanzanian National Health Research Committee (Ref: NIMR/HQ/R.8a/Vol.IX/2264), the London School of Hygiene and Tropical Medicine ethics committee (LSHTM Ref. 11,989) and the National Committee on Health Research Ethics in Denmark (Case No.1608499). This analysis was part of PhD work for BK at the Catholic University of Health and Allied Sciences (CUHAS) in Tanzania. We therefore obtained an additional approval from CUHAS/Bugando Medical Centre joint ethics and review committee.

## Results

### Baseline characteristics

A total of 1947 participants were enrolled in the study of which 655 (34%) were HIV-uninfected, 956 (49%) were ART-naive HIV-infected and 336 (17%) were HIV-infected on ART for median duration of 53 months (interquartile range (1QR) 46, 102) months). One hundred and fifty nine (47%) participants on ART were using tenofovir diproxil fumarate containing ART regimen at the time of study enrolment. Participants’ enrolment flow chart has been previously described [20] and baseline characteristics are displayed in Table 1.The mean age was 41 years (SD 12) and 59% were females. Only about 15% of enrolled participants had attained secondary or higher level of education. Cigarette smoking was common particularly in HIV-uninfected (9%) and HIV-infected ART-naive participants (12%). A total of 596 (30%) reported current alcohol use and 556 (93%) of the current drinkers were unhealthy drinkers. Most study participants (> 80%) were physically active. Although the majority of participants had normal BMI, underweight was common in HIV-infected groups (26–27%). Overall, 43% of the participants had anaemia and this was more common (60%) in ART-naive HIV-infected adults. Up to 9% of the population had *Schistosoma mansoni*, 12–28% had hypertension and the proportion with diabetes mellitus ranged from 4% in HIV-infected adults on ART to 9% in ART-naive HIV-infected adults. Out of 128 participants with diabetes, 103 (81%) were newly diagnosed. The median duration of diabetes for the 25 known diabetic patients at study enrolment was 3 years (IQR: 1, 4).

**Table 1:** Characteristics of study participants by HIV and antiretroviral therapy (ART) status^1^

Characteristic	***N*** = 1947	HIV-uninfected (***n*** = 655)	HIV-infected ART-naive (***n*** = 956)	HIV positive on ART (***n*** = 336)
Age (years); mean (SD)^2^	1947	42.2 (13.2)	38.2 (10.9)	44.8 (10.1)
18–30		139 (21.2)	256 (26.8)	25 (7.4)
31–40		183 (27.9)	342 (35.8)	98 (29.2)
41–50		171 (26.1)	223 (23.3)	127 (37.8)
> 50		162 (24.7)	135 (14.1)	86 (25.6)
Sex, female	1947	371 (56.6)	577 (60.4)	209 (62.2)
Education level	1942			
No formal education		80 (12.2)	158 (16.6)	81 (24.2)
Primary		426 (65.0)	676 (71.0)	224 (66.9)
Secondary/Tertiary		149 (22.8)	118 (12.4)	30 (9.0)
SES^3^ tertile	1942			
Lower		167 (25.5)	316 (33.2)	165 (49.3)
Middle		220 (33.6)	328 (34.5)	99 (29.6)
Upper		268 (40.9)	308 (32.4)	71 (21.2)
Smoking	1942			
Never smoked		507 (77.5)	727 (76.4)	240 (71.4)
Past smoked		89 (13.6)	116 (12.2)	81 (24.1)
Current smoker		58 (8.9)	109 (11.5)	15 (4.5)
Alcohol use	1942			
Never		212 (32.4)	248 (26.1)	93 (27.7)
Ex-drinker		231 (35.3)	376 (39.5)	186 (55.4)
Current moderate drinker		18 (2.8)	16 (1.7)	6 (1.8)
Current unhealthy drinker^4^		193 (29.5)	312 (32.8)	51 (15.2)
Antiretroviral regimen	335			
Tenofovir containing		–	–	159 (47.5)
Other regimen		–	–	176 (52.5)
Physically inactive^5^	1941	82 (12.6)	137 (14.4)	51 (15.2)
Body mass index (kg/m^2^), mean (SD)^2^	1946	23.6 (4.9)	21.1 (4.1)	20.7 (3.7)
Underweight (< 18.5)		86 (13.1)	254 (26.6)	86 (25.6)
Normal (18.5 - < 25)		351 (53.6)	565 (59.2)	214 (63.7)
Overweight/obesity (≥25)		218 (33.3)	136 (14.2)	36 (10.7)
Fat mass index (kg/m^2^), mean (SD)^2^	1900	6.3 (4.0)	4.6 (3.4)	4.5 (3.1)
Fat-free mass index (kg/m^2^)	1900	17.1 (1.9)	16.3 (1.8)	16.0 (1.6)
Haemoglobin level (g/dL), mean (SD)^2^	1942	13.5 (2.0)	11.5 (2.4)	12.6 (1.9)
Anemia^6^		127 (19.5)	577 (60.4)	124 (37.0)
*S. mansoni* egg(s) seen	1768	50 (8.5)	72 (8.2)	19 (6.2)
Systolic blood pressure (mmHg), mean (SD)^2^		125.7 (20.5)	112.6 (18.0)	110.2 (18.9)
Diastolic blood pressure (mmHg), mean (SD)^2^		81.7 (12.3)	75.6 (11.7)	74.1 (11.9)
Hypertension^7^	1943	182 (27.8)	127 (13.3)	39 (11.7)
Diabetes Mellitus^8^	1941			
Normal (≤ 7.7 mmol/L)		378 (58.1)	417 (43.7)	172 (51.3)
Prediabetes (7.8–11.0 mmol/L)		246 (37.8)	449 (47.0)	151 (45.1)
Diabetes (≥ 11.1 mmol/L)		27 (4.2)	89 (9.3)	12 (3.6)

^1^Data are number (%) unless otherwise specified and for some variables the sum may not equal to column totals due to missing values; ^2^Standard deviation; ^3^Socioeconomic status calculated using principal component analysis; ^4^Habitual alcohol drinking of more than two standard drinks for women or more than three standard drinks for men; ^5^Calculated as metabolic equivalents based on total time spent in moderate and vigorous intensity physical activity per week; ^6^Haemoglobin level < 12 mg/dL for women and < 13 mg/dL for men; ^7^Systolic blood pressure ≥ 140 mmHg and/or diastolic blood pressure ≥ 90 mmHg or on medication for hypertension; ^8^Two hour oral glucose tolerance test blood glucose level ≥ 11.1 mmol/L or on medication for diabetes

### Renal function

Table 2 describes renal function by HIV status. The results for eGFR were available for 1942 participants: 654 HIV-uninfected, 955 ART-naive HIV-infected and 333 HIV-infected on ART. The mean eGFR was highest (129.5 mL/min/1.73m^2^) in HIV-infected adults on ART followed by 111.2 mL/min/1.73m^2^ in the HIV-uninfected group and 109.7 mL/min/1.73m^2^ in ART-naive HIV-infected group. Although the HIV-infected adults on ART had a higher mean eGFR, this group also had a larger proportion of participants with severely impaired renal function i.e. moderately to severely (3%) and severely decreased (0.9%) renal function, and kidney failure (0.9%). Overall, six participants (0.3%) - three (0.3%) in the ART-naive HIV-infected group and three (0.9%) in HIV-infected adults on ART group had end stage kidney disease (eGFR < 15 mL/min/1.73m^2^). The proportion of participants with impaired renal function (eGFR < 60 mL/min/1.73m^2^) ranged from 5.7% in HIV-uninfected group to 8.1% in the ART naive HIV-infected adults. Hyperfiltration was most common (42%) in HIV-infected adults on ART and 65 (46%) out of 141 hyperfiltrating HIV-infected adults on ART were on tenofovir diproxil fumarate. Of those who were on a tenofovir containing regimen, 65 (41.1%) had hyperfiltration vs 76 (43.7%) who were on other ART regimens.

**Table 2:** Estimated glomerular filtration rate by HIV and antiretroviral therapy (ART) status

	HIV-uninfected (***n*** = 654)^**1**^	HIV-infected ART naive (***n*** = 955)^**1**^	p* value^**2**^	HIV-infected on ART (***n*** = 333)^**1**^	p* value^**3**^	p* value^**4**^
eGFR (ml/min/1.73m^2^), mean (SD)^5^	111.2 (37.6)	109.7 (35.3)	0.06	129.5 (59.6)	**< 0.001**	**< 0.001**
Impaired renal function (eGFR< 60 mL/min/1.73m^2^), n (%)	37 (5.7)	77 (8.1)	**0.045**	21 (6.3)	0.20	0.76
eGFR categories (in eGFR > 135 mL/min/1.73m^2^)						
High normal (> 135)^6^	173 (26.5)	257 (26.9)	0.73	141 (42.3)	**< 0.001**	**< 0.001**
Normal (90–135)	300 (45.9)	417 (43.7)		127 (38.1)		
Mildly decreased (60–89)	144 (22.0)	204 (21.4)		44 (13.2)		
Mildly to moderately decreased (45–59)	26 (4.0)	50 (5.2)		5 (1.5)		
Moderately to severely decreased (30–44)	8 (1.2)	17 (1.8)		10 (3.0)		
Severely decreased (15–29)	3 (0.5)	7 (0.7)		3 (0.9)		
Kidney failure (< 15)	0 (0.0)	3 (0.3)		3 (0.9)		

^1^Missing eGFR results: One in HIV-uninfected, one in ART-naive HIV-infected and three in HIV-infected on ART; ^2^Comparing ART-naive HIV-infected with HIV-uninfected group; ^3^Comparing HIV-infected on ART with ART-naive HIV-infected group; ^4^Comparing HIV-infected on ART with HIV-uninfected group; ^5^Standard deviation; ^6^Renal hyper filtration

*Adjusted for age, sex and fat-free mass

### Factors associated with eGFR and impaired renal function

Correlates of eGFR from linear regression model adjusted for age, sex and fat-free mass are presented in Table 3. Factors associated with lower eGFR were older age, higher level of education, higher socioeconomic status, unhealthy alcohol drinking, higher BMI and diabetes mellitus. Male sex and being HIV-infected on ART were associated with higher eGFR. One year increase in age was associated with 0.3 mL/min/1.73m^2^ lower eGFR (95% Confidence interval (CI):-0.4, − 0.1; *p* = 0.001). Having completed primary school was associated with 7.5 mL/min/1.73m^2^ (95% CI: − 12.8, − 2.2, *p* = 0.005) lower eGFR and those with secondary to tertiary level of education had 7.6 mL/min/1.73m^2^ (95% CI: − 14.6, − 0.7, *p* = 0.03) lower eGFR compared to no formal education group. The eGFR was lower by 7.0 mL/min/1.73m^2^ (95% CI: − 11.8, − 2.3, *p* = 0.004) in the upper socioeconomic tertile compared to lower socioeconomic tertile and unhealthy alcohol drinking was associated with 4.7 mL/min/1.73m^2^ (95% CI: − 9.0, − 0.4, *p* = 0.03) lower eGFR compared to non-alcohol drinkers. One unit increase in BMI was associated with 0.58 mL/min/1.73m^2^ (95% CI:-1.16, − 0.01, *p* = 0.047) lower eGFR and diabetes mellitus was associated with 11.2 mL/min/1.73m^2^ (95% CI: − 19.3, − 3.2, *p* = 0.006) lower eGFR compared to non diabetic. Male sex was associated with 13.0 (95% CI: 7.7, 18.2, *p* <  0.001) higher eGFR compared to females and being HIV-infected on ART was associated with 18.3 (95% CI: 12.8, 23.9, p <  0.001) higher eGFR compared to HIV-uninfected individuals. Anaemia, blood pressure and presence of *Schistosoma mansoni* eggs were not associated with eGFR.

**Table 3:** Factors associated with eGFR^1^ in HIV-infected and HIV-uninfected adults

Characteristic	n	Mean eGFR^**1**^	Adjusted coefficients^**2**^ (95% CI^**3**^)	***p*** value^**4**^	***p*** value for interaction*
Age (years)	1942	113.6	**− 0.3 (− 0.4, − 0.1)**	**0.001**	0.25
Sex					0.51
Female	1156	111.4	0		
Male	786	116.7	**13.0 (7.7, 18.2)**	**< 0.001**	
HIV status					
HIV-uninfected	654	111.2	0		
HIV-infected ART-naive	955	109.7	−4.1 (−8.4, 0.1)	0.06	
HIV-infected on ART	333	129.5	**18.3 (12.8, 23.9)**	**< 0.001**	
Education level					0.20
No formal education	317	119.1	0		
Primary	1323	112.3	**−7.5 (−12.8, −2.2)**	**0.005**	
Secondary/Tertiary	297	113.5	**−7.6 (− 14.6, −0.7)**	**0.03**	
SES^5^ tertiles					0.73
Lower	644	116.9	0		
Middle	646	114.6	−2.4 (−7.0, 2.3)	0.32	
Upper	647	109.3	**−7.0 (−11.8, −2.3)**	**0.004**	
Smoking					0.20
Never smoked	1471	112.9	0		
Past smoked	285	116.3	2.2 (−3.9, 8.3)	0.48	
Current smoker	181	115.1	−1.4 (−8.8, 5.9)	0.71	
Alcohol use					0.16
Non drinker	1342	114.6	0		
Current moderate drinker	40	121.9	7.2 (−6.1, 20.5)	0.29	
Current unhealthy drinker^6^	555	110.7	**−4.7 (−9.0, −0.4)**	**0.03**	
Antiretroviral regimen					
Tenofovir containing	174	130.4	0		
Other regimen	158	128.8	−2.7 (−15.6, 10.3)	0.69	
Physical activity (minutes/week)^7^					0.06
Active (≥ 600)	1667	113.0	0		
Inactive (< 600)	229	117.2	4.4 (−1.3, 10.0)	0.13	
Body mass index (kg/m^2^)	1941	113.6	**−0.58 (−1.16, − 0.01)**	**0.047**	0.31
Underweight (< 18.5)	423	118.6	2.9 (−2.3, 8.1)	0.28	
Normal (18.5 - < 25)	1128	114.2	0		
Overweight/obesity (≥25)	390	106.4	−3.2 (−8.6, 2.2)	0.25	
Haemoglobin (g/dL)	1938	113.5	0.3 (−0.6, 1.2)	0.46	**0.02**
Normal	1112	113.9	0		
Anaemia^8^	826	113.1	−1.9 (−5.8, 2.0)	0.34	
*S. mansoni* egg(s) seen					0.34
No	1623	113.9	0		
Yes	141	114.7	−1.2 (−8.6, 6.2)	0.76	
Systolic blood pressure (mmHg)	1938	113.7	−0.02 (−0.12, 0.08)	0.73	0.97
Diastolic blood pressure (mmHg)	1938	113.7	−0.1 (− 0.2, 0.1)	0.40	0.39
Hypertension^9^					0.91
No	1590	114.6	0		
Yes	348	109.4	−1.7 (−6.8, 3.4)	0.52	
Diabetes Mellitus^10^					0.36
Normal (≤ 7.7)	965	112.8	0		
Prediabetes (7.8–11.0)	844	115.9	3.0 (−0.9, 6.9)	0.14	
Diabetes (≥ 11.1)	128	103.5	**−10.2 (−18.2, −2.3)**	**0.01**	

^1^Estimated glomerular filtration rate (mL/min/1.73m^2)^; ^2^Adjusted for age sex and fat-free mass; ^3^Confidence interval; ^4^p-value for 2 tailored t test; ^5^Socioeconomic status calculated using principal component analysis; ^6^Habitual alcohol drinking of more than two standard drinks for women or more than three standard drinks for men; ^7^Metabolic equivalent - calculations based on total time spent in moderate and vigorous intensity physical activity per week; ^8^Haemoglobin level < 12 mg/dL for women and < 13 mg/dL for men; ^9^Systolic blood pressure ≥ 140 mmHg and/or diastolic blood pressure ≥ 90 mmHg or on medication for hypertension; ^10^Two hour oral glucose tolerance test blood glucose level ≥ 11.1 mmol/L or on medication for diabetes

*p-value (Wald test) for interaction with HIV status

Table 4 summarizes correlates of impaired renal function using logistic regression model adjusted for age sex and fat-free mass. The only factors associated with impaired renal function were diabetes mellitus and anaemia. Being anaemic was associated with almost two times higher odds of impaired renal function compared to non-anaemic individuals - adjusted odds ratio (aOR) 1.9 (95% CI: 1.3, 2.7; *p* = 0.001) and being diabetic was associated with more than twofold increase in odds of impaired renal function compared to non diabetics - aOR 2.4 (95% CI: 1.4, 4.4; *p* = 0.003). Age, sex, BMI, blood pressure, and presence of *Schistosoma mansoni* eggs were not associated with impaired renal function.

**Table 4:** Factors associated with impaired renal function in HIV-infected and HIV-uninfected adults

	n	Impaired renal function^**1**^ n (%)	aOR^**2**^ (95% CI^**3**^)	***p*** value^**4**^
Age categories (years)				
18–30	420	26 (6.2)	0	
31–40	621	49 (7.9)	1.3 (0.8, 2.1)	0.32
41–50	519	32 (6.2)	1.0 (0.6, 1.7)	0.98
> 50	382	28 (7.3)	1.2 (0.7, 2.2)	0.44
Sex				
Female	1156	82 (7.1)	0	
Male	786	53 (6.7)	1.0 (0.6, 1.6)	0.88
HIV status				
HIV-uninfected	654	37 (5.7)	0	
HIV-infected ART-naive	955	77 (8.1)	1.5 (1.0, 2.3)	0.048
HIV-infected on ART	333	21 (6.3)	1.1 (0.6, 1.9)	0.75
Education level				
No formal education	317	17 (5.4)	0	
Primary	1323	96 (7.3)	1.5 (0.9, 2.5)	0.17
Secondary/Tertiary	297	21 (7.1)	1.5 (0.7, 3.0)	0.24
SES^5^tertiles				
Lower	644	46 (7.1)	0	
Middle	646	41 (6.4)	0.9 (0.6, 1.4)	0.62
Upper	647	47 (7.3)	1.0 (0.7, 1.6)	0.95
Smoking				
Never smoked	1471	99 (6.7)	0	
Past smoked	285	22 (7.7)	1.2 (0.7, 2.1)	0.51
Current smoker	181	13 (7.2)	1.3 (0.6, 2.5)	0.51
Alcohol use				
Non drinker	1342	92 (6.9)	0	
Current moderate drinker	40	2 (5.0)	0.7 (0.2, 3.1)	0.68
Unhealthy drinker^6^	555	40 (7.2)	1.1 (0.7, 1.6)	0.61
Antiretroviral regimen				
Tenofovir containing	174	7 (4.0)	0	
Other regimen	158	13 (8.2)	2.1 (0.8, 5.6)	0.12
Physical activity (minutes/week)^7^				
Active (≥ 600)	1667	110 (6.6)	0	
Inactive (< 600)	269	24 (8.9)	1.4 (0.9, 2.2)	0.20
Body mass index (kg/m^2^)				
Underweight (< 18.5)	423	29 (6.9)	1.0 (0.6, 1.6)	0.89
Normal (18.5- < 25)	1128	79 (7.0)	0	
Overweight/obesity (≥25)	390	26 (6.7)	0.9 (0.6, 1.5)	0.76
Anaemia^8^				
No	1112	60 (5.4)	0	
Yes	826	75 (9.1)	**1.9 (1.3, 2.7)**	**0.001**
*S. mansoni*egg(s) seen				
No	1623	118 (7.3)	0	
Yes	141	8 (5.7)	0.8 (0.4, 1.7)	0.60
Hypertension^9^				
No	1590	109 (6.9)	0	
Yes	348	24 (6.9)	1.0 (0.6, 1.6)	0.95
Diabetes Mellitus^10^				
Normal (≤ 7.7)	965	61 (6.3)	0	
Prediabetes (7.8–11.0)	844	56 (6.6)	1.1 (0.7, 1.6)	0.72
Diabetes (≥ 11.1)	128	18 (14.1)	**2.3 (1.3, 4.2)**	**0.004**

^1^Estimated glomerular filtration rate < 60 mL/min/1.73m^2)^; ^2^Adjusted odds ratio - adjusted for age sex and fat-free mass index; ^3^Confidence interval; ^4^p-value for 2 tailored Wald test; ^5^Socioeconomic status calculated using principal component analysis; ^6^Habitual alcohol drinking of more than two standard drinks for women or more than three standard drinks for men; ^7^Metabolic equivalent - calculations based on total time spent in moderate and vigorous intensity physical activity per week; ^8^Haemoglobin level < 12 mg/dL for women and < 13 mg/dL for men; ^9^Systolic blood pressure ≥ 140 mmHg and/or diastolic blood pressure ≥ 90 mmHg or on medication for hypertension; ^10^Two hour oral glucose tolerance test blood glucose level ≥ 11.1 mmol/L or on medication for diabetes

### Effect modification in the relationship between HIV status and eGFR

HIV status modified the effect of haemoglobin level on eGFR (*p* value 0.02 - Table 3). With all participants combined, haemoglobin level was not associated with eGFR levels in linear regression model. However, when stratified by HIV status, one unit increase in haemoglobin was associated with − 3.4 (95%CI: − 5.2, − 1.7) lower eGFR in HIV-uninfected adults but was not associated with eGFR in HIV-infected adults. Overall, being anaemic was associated with almost twofold higher odds of impaired renal function (95% CI: 1.2, 2.7, *p* = 0.001) compared to non-anaemic individuals (Table 4) but in the stratified analysis this association was only observed in ART-naive HIV-infected group - aOR 2.1 (95% CI: 1.2, 3.6, *p* = 0.007). We present all correlates of eGFR (Additional file 1) and correlates of impaired renal function (Additional file 2) by HIV status.

## Discussion

We report a non-ignorable prevalence (7%) of impaired renal function (eGFR < 60 mL/min/1.73m^2^) in a relatively young population of HIV-infected and HIV-uninfected adults in north-western Tanzania. Moderate to severely impaired renal function was particularly common in HIV-infected adults, occurring in 2.8% of ART-naive HIV-infected adults and 4.8% of HIV-infected adults on ART. HIV-infected adults on ART had higher mean eGFR than both HIV-uninfected and ART-naive HIV-infected adults, but this difference was driven by the higher proportion of eGFR values that were > 135 mL/min/1.73m^2^ in HIV-infected adults on ART. Correlates of lower eGFR were older age, higher education level, higher socioeconomic status, unhealthy alcohol drinking and diabetes mellitus. Anaemia was associated with higher odds of impaired renal function.

To put this in context, this level of prevalence of impaired kidney function is usually seen in populations aged 50 or more years in high-income settings [28], and our study suggest that impaired kidney function has the same prevalence in people who are on average 10 years younger in SSA. Our study reports higher prevalence of impaired renal function in HIV-infected compared to HIV-uninfected adults. In line with general population-based epidemiological studies in SSA [14, 29], we found statistically significant higher prevalence of impaired renal function in ART-naive HIV-infected compared to HIV-uninfected adults; this could be due to HIV-associated nephropathy [6, 30]. In addition, compared to ART-naive HIV infected adults, HIV-infected adults on ART had lower prevalence of impaired renal function (eGFR < 60 mL/min/1.73m^2^), although the difference did not reach statistical significance. On the other hand, the prevalence of moderately-severely impaired renal function (eGFR < 45 mL/min/1.73m^2^) was higher in HIV-infected adults on ART (4.8% vs. 2.8% in ART-naive HIV-infected and 1.7% in HIV-uninfected groups). These findings are in line with existing literature where modern tenofovir-based ART has been reported to improve renal function in most patients [31, 32], while leading to progression of kidney disease in others [33–35].

The mean eGFR was higher in HIV-infected adults on ART compared to both HIV-uninfected adults and ART-naive HIV-infected adults. This difference in mean eGFR was driven by the large proportion of HIV-infected adults on ART with eGFR greater than 135 mL/min/m^2^. Of note, as is the case in most of SSA, 46% of ART-experienced HIV-infected participants with hyperfiltration were receiving ART regimens containing tenofovir diproxil fumarate. Tenofovir has been shown to cause renal hyperfiltration in African adults with HIV [34, 35]. Renal hyperfiltration, in turn, is associated with more rapid progression of kidney disease in HIV-infected adults on tenofovir [34] as well as cardiovascular disease [36]. In long run, the overall renal benefit of HIV viral suppression in improving HIV associated nephropathy could therefore be outweighed by nephrotoxic effects of tenofovir containing ART in populations at high risk for kidney disease. The significance of this finding remains unclear and deserves further investigation in prospective cohort studies.

In our study, higher socioeconomic status and higher educational achievement were associated with lower eGFR. These finding differs from reports in studies conducted in high income countries where indicators of higher socioeconomic status have been associated with higher eGFR and less impaired renal function [37–39]. Findings from the ARIC study in the United States showed that individuals with less than high school education had almost two times higher odds of impaired renal function compared to those with college education [40] and similar findings have been reported in other studies conducted in Europe [41, 42]. A recent meta-analysis confirmed the association between lower income and education with kidney disease, but noted that data are scarce in SSA [38]. Our own population based study in Tanzania previously demonstrated a link between higher socioeconomic status and kidney disease [14], a finding that we have now confirmed in this study. The association between high socioeconomic status and kidney disease in Africa could be mediated through obesity which is more common in wealthier households in Africa [43]. In our study, 33% of participants in the higher socioeconomic status were overweight or obese compared to about 10% in the lower socioeconomic status group, and almost all overweight or obese participants were females and females had lower eGFR compared to males.

Diabetes mellitus was associated with lower eGFR and with higher odds of impaired renal function. These findings are consistent with available body of literature globally. Diabetes mellitus typically leads to hyperfiltration and then progresses to reduced eGFR. This association between diabetes and reduced eGFR in our study is notable, because nearly 81% of study participants were newly diagnosed with diabetes mellitus at the time of study enrolment. Very often, diabetes mellitus is an asymptomatic condition and may go unnoticed in many people until they present with chronic complications. Therefore, it appears likely that the diabetes mellitus may have been present but undiagnosed for many years prior to study enrolment. This is typically true in SSA context where health systems are not always well-equipped for NCD care including diabetes mellitus [44, 45] and routine screening for diabetes is rarely done [46].

Anemia was associated with almost twofold higher odds of impaired renal function compared to no anemia. These findings are in line with medical literature. Kidney disease can cause anemia due to reduced levels of erythropoietin levels [47]. Interestingly, the relationship between haemoglobin level and eGFR differed by HIV status.

As with any study, our study has limitations. Given the cross-sectional design, it is not possible to establish causal link between exposures of interest and impaired renal function. In addition, participants recruited from NUSTART and TBNUT cohorts largely represent survivors due to high mortality rate during the original studies [19] and therefore our estimates for impaired renal function and observed risk profile could be influenced by survival bias.

## Conclusion and recommendations

Impaired renal function is high in the study population especially in ART-naive HIV-infected adults. Interventions for prevention of impaired renal function are needed in the study population with special focus in HIV-infected adults and those with high socioeconomic status. Interventions on modifiable risk factors such as alcohol and weight reduction are warranted in this population. Future research should focus on the consequences of hyperfiltration in HIV-infected adults on tenofovir-containing ART.

## Supplementary Information

**Additional file 1.** Factors associated with estimated glolerular filtration rate by HIV status. Data description: The additional file 1 contains supplementary tables 1 - 3 describing factors associated with eGFR stratified by HIV-status

**Additional file 2.** Factors associated with impaired renal function (eGFR< 60/mL/1.73m^2^) by HIV status. Data description: The additional file 2 contains supplementary Tables 4–6 describing factors associated with impaired renal function (eGFR< 60/mL/1.73m^2^) by HIV-status

## Data Availability

The Medical Research Coordinating Committee (MRCC) of the National Institute for Medical Research (NIMR) in Tanzania does not allow data to be transferred or shared without their permission. Therefore data will be available upon request and approval by the Tanzanian MRCC. Researchers who meet the criteria to access confidential data may request access to the data through the following contact details: The MRCC secretariat, National Institute for Medical Research, 2448, Baraka Obama Road, P O Box 9653, Dar es Salaam, Tanzania. Email address: ethics@nimr.or.tz

## Abbreviations

aOR: Adjusted odds ratio
BP: Blood Pressure
ART: Antiretroviral therapy
BMI: Body mass index
CICADA: Chronic Infections, Co-morbidities and Diabetes in Africa
CKD-EPI: Chronic Kidney Disease Epidemiology
CUHAS: Catholic University of Health and Allied Sciences
eGFR: Estimated glomerular filtration rate
HIV: Human immunodeficiency virus
1QR: Interquartile range
KDIGO: Kidney Disease Improving Global Outcome
NCD: Non-communicable disease
NIMR: National Institute for Medical Research
NUSTART: Nutritional Support for African Adults Starting Antiretroviral Therapy
OGTT: Oral glucose tolerance test
SES: Socioeconomic status
SSA: Sub-Saharan Africa
SD: Standard deviation
STEPS: STEPwise approach to chronic disease risk factor surveillance
TB-NUT: Nutrition, Diabetes and Pulmonary Tuberculosis
WHO: World Health Organization
