# Supplementary material for: Risk factors for impaired renal function in HIV-infected and HIV-uninfected adults: cross-sectional study in North-Western Tanzania
Source: BMC Nephrol. 2021 Oct 29;22:355. doi: 10.1186/s12882-021-02563-z (PMC8555220; doi:10.1186/s12882-021-02563-z)
Supplement: Supplementary file 1 — Additional file 1. Factors associated with estimated glolerular filtration rate by HIV status. Data description: The additional file 1 contains supplementary tables 1 - 3 describing factors associated with eGFR stratified by HIV-status [file 12882_2021_2563_MOESM1_ESM.docx]

**Factors associated with estimated glolerular filtration rate by HIV status**

**Supplementary table 1: Factors associated with eGFR^1^ in HIV-infected adults on antiretroviral therapy**

| **Characteristic** | **n = 333** | **Mean eGFR^1^** | **Adjusted coefficients^2^ (95% CI^3^)** | **p value^4^** |
| --- | --- | --- | --- | --- |
| Age (years) | 333 | 129.5 | **-0.9 (-1.5, -0.2)** | **0.01** |
| Sex |  |  |  |  |
| Female | 208 | 124.1 | 0 |  |
| Male | 125 | 138.4 | **24.2 (6.3, 42.2)** | **0.008** |
| Education level |  |  |  |  |
| No formal education | 79 | 139.7 | 0 |  |
| Primary | 223 | 124.6 | **-18.1 (-33.7, -2.4)** | **0.02** |
| Secondary/Tertiary | 30 | 137.0 | -5.7 (-31.0, 19.6) | 0.66 |
| SES^5^ tertiles |  |  |  |  |
| Lower | 162 | 130.3 | 0 |  |
| Middle | 99 | 132.2 | 0.3 (-14.9, 15.5) | 0.97 |
| Upper | 71 | 123.3 | -6.5 (-23.7, 10.7) | 0.46 |
| Smoking |  |  |  |  |
| Never smoked | 239 | 130.3 | 0 |  |
| Past smoked | 80 | 127.3 | -12.6 (-31.0, 5.8) | 0.18 |
| Current smoker | 14 | 128.6 | -15.9 (-51.3, 19.6) | 0.38 |
| Alcohol use |  |  |  |  |
| Non drinker | 277 | 127.5 | 0 |  |
| Current moderate drinker | 6 | 133.2 | 1.4 (-47.0, 49.9) | 0.95 |
| Current unhealthy drinker^6^ | 50 | 139.9 | 9.7 (-9.2, 28.7) | 0.31 |
| Antiretroviral regimen |  |  |  |  |
| Tenofovir containing | 179 | 130.4 | 0 |  |
| Other regimen | 156 | 128.8 | -2.7 (-15.6, 10.3) | 0.69 |
| Physical activity (minutes/week)^7^ |  |  |  |  |
| Active (≥ 600) | 282 | 127.1 | 0 |  |
| Inactive (< 600) | 51 | 142.5 | 17.0 (-1.1, 35.1) | 0.07 |
| Body mass index (kg/m^2^) | 333 | 129.5 | -0.6 (-2.9, 1.7) | 0.60 |
| Underweight (<18.5) | 85 | 130.1 | -3.3 (-19.6, 13.0) | 0.69 |
| Normal (18.5 - <25) | 212 | 131.0 | 0 |  |
| Overweight/obesity (≥25) | 36 | 118.6 | -0.2 (-23.7, 23.3) | 0.99 |
| Haemoglobin level (g/dL) | 332 | 129.5 | -0.3 (-4.1, 3.5) | 0.88 |
| Normal | 210 | 128.7 | 0 |  |
| Anemia^8^ | 122 | 130.8 | 2.9 (-10.7, 16.6) | 0.67 |
| *S. mansoni* egg(s) seen |  |  |  |  |
| No | 283 | 131.3 | 0 |  |
| Yes | 19 | 128.4 | -4.1 (-33.4, 25.2) | 0.78 |
| Systolic blood pressure (mmHg) | 331 | 129.9 | 0.1 (-0.3, 0.4) | 0.71 |
| Diastolic blood pressure (mmHg) | 331 | 129.9 | -0.1 (-0.7, 0.4) | 0.62 |
| Hypertension^9^ |  |  |  |  |
| No | 292 | 130.3 | 0 |  |
| Yes | 39 | 126.4 | 0.8 (-19.5, 21.2) | 0.94 |
| Diabetes Mellitus^10^ |  |  |  |  |
| Normal (≤ 7.7) | 171 | 125.4 | 0 |  |
| Prediabetes (7.8 - 11.0) | 149 | 134.4 | 10.4 (-2.8, 23.6) | 0.12 |
| Diabetes (≥ 11.1) | 12 | 123.7 | 4.4 (-31.9, 40.8) | 0.81 |

^1^Estimated glomerular filtration rate (mL/min/1.73m^2)^; ^2^Adjusted for age sex and fat-free mass; ^3^Confidence interval; ^4^p-value for 2 tailored t test; ^5^Socioeconomic status calculated using principal component analysis; ^6^Habitual alcohol drinking of more than two standard drinks for women or more than three standard drinks for men; ^7^Metabolic equivalent - calculations based on total time spent in moderate and vigorous intensity physical activity per week; ^8^Haemoglobin level <12mg/dL for women and <13mg/dL for men; ^9^Systolic blood pressure ≥140 mmHg and/or diastolic blood pressure ≥90 mmHg or on medication for hypertension; ^10^Two hour oral glucose tolerance test blood glucose level ≥ 11.1mmol/L or on medication for diabetes

**Supplementary table 2: Factors associated with eGFR^1^ in antiretroviral therapy-naive HIV-infected adults**

| **Characteristic** | **n = 955** | **Mean eGFR^1^** | **Adjusted coefficients^2^ (95% CI^3^)** | **p value^4^** |
| --- | --- | --- | --- | --- |
| Age (years) | 955 | 109.7 | **-0.4 (-0.6, -0.1)** | **0.001** |
| Sex |  |  |  |  |
| Female | 577 | 108.9 | 0 |  |
| Male | 378 | 110.8 | **7.7 (1.1, 14.2)** | **0.02** |
| Education level |  |  |  |  |
| No formal education | 158 | 112.15 | 0 |  |
| Primary | 675 | 109.41 | -3.0 (-9.3, 3.3) | 0.35 |
| Secondary/Tertiary | 118 | 108.50 | -5.3 (-14.2, 3.5) | 0.23 |
| SES^5^ tertiles |  |  |  |  |
| Lower | 316 | 111.4 | 0 |  |
| Middle | 327 | 109.9 | -1.3 (-6.8, 4.3) | 0.66 |
| Upper | 308 | 107.9 | -2.7 (-8.44, 3.0) | 0.35 |
| Smoking |  |  |  |  |
| Never smoked | 726 | 109.7 | 0 |  |
| Past smoked | 116 | 107.8 | -1.8 (-9.5, 6.0) | 0.66 |
| Current smoker | 109 | 111.0 | -0.3 (-8.5, 7.9) | 0.94 |
| Alcohol use |  |  |  |  |
| Non drinker | 623 | 111.0 | 0 |  |
| Current moderate drinker | 16 | 122.3 | -10.0 (-7.4, 2 7.4) | 0.26 |
| Current unhealthy drinker^6^ | 312 | 106.2 | **-5.2 (-10.2, -0.3)** | **0.04** |
| Physical activity (minutes/week) ^7^ |  |  |  |  |
| Active (≥ 600) | 815 | 109.2 | 0 |  |
| Inactive (< 600) | 136 | 112.6 | 4.4 (-2.3, 11.2) | 0.20 |
| Body mass index (kg/m^2^) | 954 | 109.7 | -0.2 (-0.9, 0.6) | 0.67 |
| Underweight (<18.5) | 253 | 113.5 | 4.4 (-1.6, 10.44) | 0.15 |
| Normal (18.5 - <25) | 565 | 108.9 | 0 |  |
| Overweight/obesity (≥25) | 136 | 106.0 | 1.4 (-6.0, 8.8) | 0.71 |
| Haemoglobin level (g/dL) | 332 | 129.5 | 1.00 (-0.03, 2.04) | 0.06 |
| Normal | 210 | 128.7 | 0 |  |
| Anemia^8^ | 122 | 130.8 | -3.4 (-8.1, 1.3) | 0.15 |
| *S. mansoni* egg(s) seen |  |  |  |  |
| No | 802 | 109.2 | 0 |  |
| Yes | 72 | 115.0 | 4.6 (-4.0, 13.1) | 0.29 |
| Systolic blood pressure (mmHg) | 953 | 109.7 | 0.0 (-0.1, 0.2) | 0.67 |
| Diastolic blood pressure (mmHg) | 953 | 109.7 | 0.1 (-0.1, 0.3) | 0.34 |
| Hypertension^9^ |  |  |  |  |
| No | 826 | 110.5 | 0 |  |
| Yes | 127 | 104.5 | -1.7 (-8.7, 5.2) | 0.63 |
| Diabetes Mellitus^10^ |  |  |  |  |
| Normal (≤ 7.7) | 417 | 110.6 | 0 |  |
| Prediabetes (7.8 - 11.0) | 449 | 110.2 | -0.1 (-4.9, 4.6) | 0.96 |
| Diabetes (≥ 11.1) | 89 | 102.5 | **-10.0 (-18.5, -1.5)** | **0.02** |

^1^Estimated glomerular filtration rate (mL/min/1.73m^2)^; ^2^Adjusted for age sex and fat-free mass; ^3^Confidence interval; ^4^p-value for 2 tailored t test; ^5^Socioeconomic status calculated using principal component analysis; ^6^Habitual alcohol drinking of more than two standard drinks for women or more than three standard drinks for men; ^7^Metabolic equivalent - calculations based on total time spent in moderate and vigorous intensity physical activity per week; ^8^Haemoglobin level <12mg/dL for women and <13mg/dL for men; ^9^Systolic blood pressure ≥140 mmHg and/or diastolic blood pressure ≥90 mmHg or on medication for hypertension; ^10^Two hour oral glucose tolerance test blood glucose level ≥ 11.1mmol/L or on medication for diabetes

**Supplementary table 3: Factors associated with eGFR^1^ in HIV-uninfected adults**

| **Characteristic** | **n = 654** | **Mean eGFR^1^** | **Adjusted coefficients^2^ (95% CI^3^)** | **p value^4^** |
| --- | --- | --- | --- | --- |
| Age (years) | 654 | 111.2 | **-0.3 (-0.6, -0.1)** | **0.003** |
| Sex |  |  |  |  |
| Female | 371 | 108.2 | 0 |  |
| Male | 283 | 115.2 | **14.5 (6.6, 22.3)** | **<0.001** |
| Education level |  |  |  |  |
| No formal education | 80 | 112.4 | 0 |  |
| Primary | 425 | 110.5 | -4.1 (-13.4, 5.1) | 0.38 |
| Secondary/Tertiary | 149 | 112.6 | -4.6 (-15.5, 6.2) | 0.40 |
| SES^5^ tertiles |  |  |  |  |
| Lower | 166 | 114.4 | 0 |  |
| Middle | 220 | 113.6 | -1.5 (-9.2, 6.2) | 0.70 |
| Upper | 268 | 107.2 | **-8.31 (-15.9, -0.7)** | **0.03** |
| Smoking | 89 |  |  |  |
| Never smoked | 506 | 109.2 | 0 |  |
| Past smoked | 89 | 117.4 | 9.4 (-0.3, 19.1) | 0.06 |
| Current smoker | 58 | 119.4 | 9.0 (-2.5, 20.5) | 0.12 |
| Alcohol use |  |  |  |  |
| Non drinker | 442 | 111.4 | 0 |  |
| Current moderate drinker | 18 | 117.7 | -6.9 (-11.3, 25.1) | 0.46 |
| Current unhealthy drinker^6^ | 193 | 110.3 | -1.9 (-8.4, 4.7) | 0.58 |
| Physical activity (minutes/week)^7^ |  |  |  |  |
| Active (≥ 600) | 570 | 111.6 | 0 |  |
| Inactive (< 600) | 82 | 109.0 | -3.7 (-13.0, 5.6) | 0.44 |
| Body mass index (kg/m^2^) | 654 | 111.2 | -0.8 (-1.6, 0.1) | 0.08 |
| Underweight (<18.5) | 85 | 122.1 | 8.4 (-1.2, 18.0) | 0.09 |
| Normal (18.5 - <25) | 351 | 112.6 | 0 |  |
| Overweight/obesity (≥25) | 218 | 104.7 | -4.7 (-11.9, 2.5) | 0.20 |
| Haemoglobin level (g/dL) | 651 | 111.2 | **-3.4 (-5.2, -1.7)** | **<0.001** |
| Normal | 524 | 110.0 | 0 |  |
| Anemia^8^ | 127 | 116.0 | 6.9 (-0.6, 14.4) | 0.07 |
| *S.mansoni* egg(s) seen |  |  |  |  |
| No | 538 | 111.8 | 0 |  |
| Yes | 50 | 109.2 | -6.9 (-18.2, 4.3) | 0.22 |
| Systolic blood pressure (mmHg) | 654 | 111.2 | 0.0 (-0.1, 0.2) | 0.75 |
| Diastolic blood pressure (mmHg) | 654 | 111.2 | -0.1 (-0.3, 0.2) | 0.57 |
| Hypertension^9^ |  |  |  |  |
| No | 472 | 112.0 | 0 |  |
| Yes | 182 | 109.1 | 0.4 (-6.4, 7.3) | 0.90 |
| Diabetes Mellitus^10^ |  |  |  |  |
| Normal (≤ 7.7) | 377 | 109.5 | 0 |  |
| Prediabetes (7.8 - 11.0) | 246 | 114.9 | 5.8 (-0.2, 11.9) | 0.06 |
| Diabetes (≥ 11.1) | 27 | 102.3 | -5.2 (-20.1, 9.7) | 0.50 |

^1^Estimated glomerular filtration rate (mL/min/1.73m^2)^; ^2^Adjusted for age sex and fat-free mass; ^3^Confidence interval; ^4^p-value for 2 tailored t test; ^5^Socioeconomic status calculated using principal component analysis; ^6^Habitual alcohol drinking of more than two standard drinks for women or more than three standard drinks for men; ^7^Metabolic equivalent - calculations based on total time spent in moderate and vigorous intensity physical activity per week; ^8^Haemoglobin level <12mg/dL for women and <13mg/dL for men; ^9^Systolic blood pressure ≥140 mmHg and/or diastolic blood pressure ≥90 mmHg or on medication for hypertension; ^10^Two hour oral glucose tolerance test blood glucose level ≥ 11.1mmol/L or on medication for diabetes
