# Supplementary material for: Risk factors for impaired renal function in HIV-infected and HIV-uninfected adults: cross-sectional study in North-Western Tanzania
Source: BMC Nephrol. 2021 Oct 29;22:355. doi: 10.1186/s12882-021-02563-z (PMC8555220; doi:10.1186/s12882-021-02563-z)
Supplement: Supplementary file 2 — Additional file 2. Factors associated with impaired renal function (eGFR< 60/mL/1.73m2) by HIV status. Data description: The additional file 2 contains supplementary Tables 4–6 describing factors associated with impaired renal function (eGFR< 60/mL/1.73m2) by HIV-status [file 12882_2021_2563_MOESM2_ESM.docx]

**Factors associated with impaired renal function (eGFR<60/mmL/1.73m^2^) by HIV status**

**Supplementary table 4: Factors associated with impaired renal function in HIV-infected adults on antiretroviral therapy**

|  | **n** | **Impaired renal function^1^ n (%)** | **aOR^2^ (95% CI^3^)** | **p value^4^** |
| --- | --- | --- | --- | --- |
| Age categories (years) |  |  |  |  |
| 18 - 30 | 25 | 1 (4.0) | 0 |  |
| 31 - 40 | 98 | 7 (7.1) | 2.2 (0.3, 19.0) | 0.70 |
| 41 - 50 | 125 | 6 (4.8) | 1.4 (0.2, 12.4) | 0.75 |
| >50 | 85 | 7 (8.2) | 2.7 (0.3, 23.1) | 0.37 |
| Sex |  |  |  |  |
| Female | 208 | 15 (7.2) | 0 |  |
| Male | 125 | 6 (4.8) | 1.0 (0.3, 3.4) | 0.94 |
| Education level |  |  |  |  |
| No formal education | 79 | 3 (3.8) | 0 |  |
| Primary | 223 | 18 (8.1) | 3.1 (0.8, 11.4) | 0.09 |
| Secondary/Tertiary | 30 | 0 (0) | - | - |
| SES^4^ tertiles |  |  |  |  |
| Lower | 162 | 10 (6.2) | 0 |  |
| Middle | 99 | 6 (6.1) | 1.1 (0.4, 3.3) | 0.81 |
| Upper | 71 | 5 (7.0) | 1.5 (0.5, 4.7) | 0.51 |
| Smoking |  |  |  |  |
| Never smoked | 239 | 13 (5.4) | 0 |  |
| Past smoked | 80 | 7 (8.8) | 2.4 (0.7, 8.1) | 0.14 |
| Current smoker | 14 | 1 (7.1) | 2.5 (0.2, 26.0) | 0.43 |
| Alcohol use |  |  |  |  |
| Non drinker | 277 | 18 (6.5) | 0 |  |
| Current moderate drinker | 6 | 0 (0.0) | - | - |
| Unhealthy drinker^6^ | 50 | 3 (6.0) | 1.2 (0.3, 4.6) | 0.78 |
| Antiretroviral regimen |  |  |  |  |
| Tenofovir containing | 174 | 7 (4.0) | 0 |  |
| Other regimen | 158 | 13 (8.2) | 2.1 (0.8, 5.6) | 0.12 |
| Physical activity (minutes/week)^7^ |  |  |  |  |
| Active (≥ 600) | 282 | 16 (5.7) | 0 |  |
| Inactive (< 600) | 51 | 5 (9.8) | 1.7 (0.6, 5.0) | 0.35 |
| Body mass index (kg/m^2^) |  |  |  |  |
| Underweight (<18.5) | 85 | 7 (8.2) | 1.3 (0.4, 3.7) | 0.64 |
| Normal (18.5-<25) | 212 | 12 (5.7) | 0 |  |
| Overweight/obesity (≥25) | 36 | 2 (5.6) | 0.9 (0.2, 4.9) | 0.90 |
| Anaemia^8^ |  |  |  |  |
| No | 210 | 11 (5.2) | 0 |  |
| Yes | 122 | 10 (8.2) | 1.6 (0.7, 4.0) | 0.29 |
| *S. mansoni* egg(s) seen |  |  |  |  |
| No | 283 | 20 (7.1) | 0 |  |
| Yes | 19 | 0 (0.0) | - | - |
| Hypertension^9^ |  |  |  |  |
| No | 292 | 18 (6.2) | 0 |  |
| Yes | 39 | 2 (5.1) | 0.8 (0.2, 3.6) | 0.76 |
| Diabetes Mellitus^10^ |  |  |  |  |
| Normal (≤ 7.7) | 171 | 8 (4.7) | 0 |  |
| Prediabetes (7.8 - 11.0) | 149 | 13 (8.7) | 1.9 (0.8, 4.8) | 0.17 |
| Diabetes (≥ 11.1) | 12 | 0 (0.0) | - | - |

^1^Estimated glomerular filtration rate <60 mL/min/1.73m^2)^; ^2^Adjusted odds ratio - adjusted for age sex and fat-free mass index; ^3^Confidence interval; ^4^p-value for 2 tailored Wald test; ^5^Socioeconomic status calculated using principal component analysis; ^6^Habitual alcohol drinking of more than two standard drinks for women or more than three standard drinks for men; ^7^Metabolic equivalent - calculations based on total time spent in moderate and vigorous intensity physical activity per week; ^8^Haemoglobin level <12mg/dL for women and <13mg/dL for men; ^9^Systolic blood pressure ≥140 mmHg and/or diastolic blood pressure ≥90 mmHg or on medication for hypertension; ^10^Two hour oral glucose tolerance test blood glucose level ≥ 11.1mmol/L or on medication for diabetes

**Supplementary table 5: Factors associated impaired renal function in antiretroviral therapy-naive HIV-infected adults**

|  | **n** | **Impaired renal function^1^ n (%)** | **aOR^2^ (95% CI^3^)** | **p value^4^** |  |
| --- | --- | --- | --- | --- | --- |
| Age categories (years) |  |  |  |  |  |
| 18 - 30 | 256 | 18 (7.0) | 0 |  |  |
| 31 - 40 | 341 | 31 (9.1) | 1.3 (0.7, 2.3) | 0.44 |  |
| 41 - 50 | 223 | 16 (7.2) | 0.9 (0.5, 1.9) | 0.88 |  |
| >50 | 135 | 12 (8.9) | 1.3 (0.6, 2.8) | 0.55 |  |
| Sex |  |  |  |  |  |
| Female | 577 | 41 (7.1) | 0 |  |  |
| Male | 378 | 36 (9.5) | 1.3 (0.6, 2.4) | 0.50 |  |
| Education level |  |  |  |  |  |
| No formal education | 158 | 10 (6.3) | 0 |  |  |
| Primary | 675 | 56 (8.3) | 1.3 (0.6, 2.7) | 0.45 |  |
| Secondary/Tertiary | 118 | 10 (8.5) | 1.4 (0.5, 3.6) | 0.51 |  |
| SES^5^ tertiles |  |  |  |  |  |
| Lower | 316 | 28 (8.9) | 0 |  |  |
| Middle | 327 | 24 (7.3 | 0.8 (0.5, 1.4) | 0.44 |  |
| Upper | 308 | 24 (7.8) | 0.8 (0.4, 1.5) | 0.48 |  |
| Smoking |  |  |  |  |  |
| Never smoked | 726 | 53 (7.3) | 0 |  |  |
| Past smoked | 116 | 14 (12.1) | 1.5 (0.7, 3.0) | 0.31 |  |
| Current smoker | 109 | 9 (8.3) | 1.1 (0.5, 2.5) | 0.85 |  |
| Alcohol use |  |  |  |  |  |
| Non drinker | 623 | 50 (8.0) | 0 |  |  |
| Current moderate drinker | 16 | 1 (6.3) | 0.8 (0.1, 6.1) | 0.81 |  |
| Unhealthy drinker^6^ | 312 | 25 (8.0) | 1.0 (0.6, 1.6) | 0.92 |  |
| Physical activity (minutes/week)^7^ |  |  |  |  |  |
| Active (≥ 600) | 815 | 63 (7.7) | 0 |  |  |
| Active (< 600) | 136 | 13 (9.6) | 1.1 (0.6, 2.2) | 0.73 |  |
| Body mass index (kg/m^2^) |  |  |  |  |  |
| Underweight (<18.5) | 253 | 19 (7.5) | 0.9 (0.5, 1.7) | 0.71 |  |
| Normal (18.5-<25) | 565 | 49 (8.7) | 0 |  |  |
| Overweight/obesity (≥25) | 136 | 8 (5.9) | 0.6 (0.3, 1.5) | 0.32 |  |
| Anaemia^8^ |  |  |  |  |  |
| No | 378 | 21 (5.6) |  |  |  |
| Yes | 577 | 56 (9.7) | **2.1 (1.2, 3.6)** | **0.007** |  |
| *S. manson* egg(s) seen |  |  |  |  |  |
| No | 802 | 68 (8.5) | 0 |  |  |
| Yes | 72 | 6 (6.9) | 0.8 (0.3, 2.1) | 0.69 |  |
| Hypertension^9^ |  |  |  |  |  |
| No | 826 | 63 (7.6) | 0 |  |  |
| Yes | 127 | 13 (10.2) | 1.3 (0.7, 2.6) | 0.40 |  |
| Diabetes Mellitus^10^ |  |  |  |  |  |
| Normal (≤ 7.7) | 417 | 30 (7.2) | 0 |  |  |
| Prediabetes (7.8 - 11.0) | 449 | 31 (6.9) | 1.0 (0.6, 1.7) | 0.95 |  |
| Diabetes (≥ 11.1) | 89 | 16 (18.0) | **2.8 (1.4, 5.6)** | **0.004** |  |

^1^Estimated glomerular filtration rate <60 mL/min/1.73m^2)^; ^2^Adjusted odds ratio - adjusted for age, sex and fat-free mass index; ^3^Confidence interval; ^4^p-value for 2 tailored Wald test; ^5^Socioeconomic status calculated using principal component analysis; ^6^Habitual alcohol drinking of more than two standard drinks for women or more than three standard drinks for men; ^7^Metabolic equivalent - calculations based on total time spent in moderate and vigorous intensity physical activity per week; ^8^Haemoglobin level <12mg/dL for women and <13mg/dL for men; ^9^Systolic blood pressure ≥140 mmHg and/or diastolic blood pressure ≥90 mmHg or on medication for hypertension; ^10^Two hour oral glucose tolerance test blood glucose level ≥ 11.1mmol/L or on medication for diabetes

**Supplementary table 6: Factors associated with impaired renal function in HIV-uninfected adults**

|  | **n** | **Impaired renal function^1^ n (%)** | **aOR^2^ (95% CI^3^)** | **p value^4^** |
| --- | --- | --- | --- | --- |
| Age categories (years) |  |  |  |  |
| 18 - 30 | 139 | 7 (5.0 | 0 |  |
| 31 - 40 | 182 | 11 (6.0) | 1.3 (0.5, 3.4) | 0.64 |
| 41 - 50 | 171 | 10 (5.9) | 1.3 (0.5, 3.6) | 0.58 |
| >50 | 162 | 9 (5.6) | 1.3 (0.5, 3.7) | 0.58 |
| Sex |  |  |  |  |
| Female | 371 | 26 (7.0) | 0 |  |
| Male | 283 | 11 (3.9) | 0.5 (0.2, 1.3) | 0.14 |
| Education level |  |  |  |  |
| No formal education | 80 | 4 (5.0) | 0 |  |
| Primary | 425 | 22 ( 5.2) | 1.3 (0.4, 3.9) | 0.70 |
| Secondary/Tertiary | 149 | 11 (7.4) | 2.0 (0.6, 7.3) | 0.27 |
| SES^5^ tertiles |  |  |  |  |
| Lower | 166 | 8 (4.8) | 0 |  |
| Middle | 220 | 11 (5.0) | 1.1 (0.4, 2.8) | 0.89 |
| Upper | 268 | 18 (6.7) | 1.5 (0.6, 3.6) | 0.37 |
| Smoking |  |  |  |  |
| Never smoked | 506 | 33 (6.5) | 0 |  |
| Past smoked | 89 | 1 (1.1) | 0.2 (0.0, 1.5) | 0.12 |
| Current smoker | 58 | 3 (5.2) | 1.0 (0.3, 4.0) | 0.99 |
| Alcohol use |  |  |  |  |
| Non drinker | 442 | 24 (5.4) | 0 |  |
| Current moderate drinker^6^ | 18 | 1 (5.6) | 1.1 (0.1, 9.9) | 0.91 |
| Unhealthy drinker | 193 | 12 (6.2) | 1.3 (0.6, 2.8) | 0.46 |
| Physical activity (minutes/week)^7^ |  |  |  |  |
| ≥ 600 | 570 | 31 (5.4) | 0 |  |
| < 600 | 82 | 6 (7.3) | 1.9 (0.7, 5.2) | 0.20 |
| Body mass index (kg/m^2^) |  |  |  |  |
| Underweight (<18.5) | 85 | 3 (3.5) | 0.6 (0.2, 2.4) | 0.50 |
| Normal (18.5-<25) | 351 | 18 (5.1) | 0 (ref) |  |
| Overweight/obesity (≥25) | 218 | 16 (7.3) | 1.3 (.61, 3.0) | 0.47 |
| Anaemia^8^ |  |  |  |  |
| No | 524 | 28 (5.3) | 0 |  |
| Yes | 127 | 9 (7.1) | 1.2 (0.5, 2.7) | 0.66 |
| *S.manson* egg(s) seen |  |  |  |  |
| No | 538 | 30 (5.6) | 0 |  |
| Yes | 50 | 3 (6.0) | 1.5 (0.4, 5.1) | 0.56 |
| Hypertension^9^ |  |  |  |  |
| No | 472 | 28 (5.9) | 0 |  |
| Yes | 182 | 9 (5.0) | 0.8 (0.3, 1.7) | 0.51 |
| Diabetes Mellitus^10^ |  |  |  |  |
| Normal (≤ 7.7) | 377 | 23 (6.1) | 0 |  |
| Prediabetes (7.8 - 11.0) | 246 | 12 (4.9) | 0.8 (0.4, 1.7) | 0.56 |
| Diabetes (≥ 11.1) | 27 | 2 (7.4) | 1.4 (0.3, 6.5) | 0.69 |

^1^Estimated glomerular filtration rate <60 mL/min/1.73m^2)^; ^2^Adjusted odds ratio - adjusted for age, sex and fat-free mass index; ^3^Confidence interval; ^4^p-value for 2 tailored Wald test; ^5^Socioeconomic status calculated using principal component analysis; ^6^Habitual alcohol drinking of more than two standard drinks for women or more than three standard drinks for men; ^7^Metabolic equivalent - calculations based on total time spent in moderate and vigorous intensity physical activity per week; ^8^Haemoglobin level <12mg/dL for women and <13mg/dL for men; ^9^Systolic blood pressure ≥140 mmHg and/or diastolic blood pressure ≥90 mmHg or on medication for hypertension; ^10^Two hour oral glucose tolerance test blood glucose level ≥ 11.1mmol/L or on medication for diabetes
